# p16^INK4a^ Expression and Immunologic Aging in Chronic HIV Infection

**DOI:** 10.1371/journal.pone.0166759

**Published:** 2016-11-18

**Authors:** Susan Pereira Ribeiro, Jeffrey M. Milush, Edecio Cunha-Neto, Esper G. Kallas, Jorge Kalil, Luiz Felipe D. Passero, Peter W. Hunt, Steven G. Deeks, Douglas F. Nixon, Devi SenGupta

**Affiliations:** 1 Laboratory of Clinical Immunology and Allergy-LIM60/ University of Sao Paulo School of Medicine, São Paulo, Brazil; 2 Institute of Investigation in Immunology–iii-INCT, São Paulo, Brazil; 3 Department of Pathology, Case Western Reserve University, Cleveland, Ohio, United States of America; 4 Division of Experimental Medicine, Department of Medicine, University of California San Francisco, San Francisco, California, United States of America; 5 Laboratory of Immunology, Heart Institute, University of Sao Paulo School of Medicine, São Paulo, Brazil; 6 Butantan Institute, Butantan, São Paulo, SP, Brazil; 7 São Vicent Unit, Paulista Coastal Campus, São Paulo State University "Julio de Mesquita Filho", Sao Paulo, Brazil; 8 HIV/AIDS Division, Department of Medicine, San Francisco General Hospital, University of California, San Francisco, California, United States of America; 9 Department of Microbiology, Immunology & Tropical Medicine, The George Washington University, Washington DC, United States of America; University of Pittsburgh Centre for Vaccine Research, UNITED STATES

## Abstract

Chronic HIV infection is characterized by increased immune activation and immunosenescence. p16 ^**INK4a**^ (p16) is a member of the cyclin-dependent kinase antagonist family that inhibits cellular proliferation, and its protein expression increases during normal chronological aging. However, some infectious diseases can increase the expression of this anti-proliferative protein, potentially accelerating immunological aging and dysfunction. In order to investigate the immunological aging in HIV patients, p16 protein expression was evaluated by flow cytometry, in T cell subsets in a cohort of chronically HIV-infected patients on and off ART as well as age-matched healthy controls. Results showed that untreated HIV-infected subjects exhibited increased per-cell p16 protein expression that was discordant with chronological aging. ART restored p16 protein expression to levels comparable with HIV-negative subjects in the CD4 compartment, but not in CD8 T cells, which can be an indicative of an irreversible activation/exhaustion status on these cells. Additionally, the frequency of activated CD4+ and CD8+ T cells was positively correlated with p16 expression in CD4+ and CD8+ T cells in untreated subjects. In contrast to healthy controls, untreated HIV-infected individuals had increased p16 levels within the effector memory (T_EM_) subset, indicating a possible role for this marker in impaired clonal expansion during antiviral effector function. Taken together, these data demonstrate that chronic HIV infection is associated with elevated expression of the cellular aging marker p16 in T cells. ART restored normal p16 levels in the CD4+ T cell compartment, indicating that use of therapy can be of fundamental importance to normal cell cycling and maintaining immune homeostasis.

## Introduction

Despite effective antiretroviral therapy (ART), individuals infected with HIV remain at higher risk of mortality and morbidities commonly associated with aging than the general population [1]. This is in part because of the persistent viral replication and repeated stimulation of HIV-specific T cells that gradually drive these cells to senescence, characterized by exhaustion of their replicative capacity and even resulting in the loss of certain anti-HIV T cell clones (reviewed in [2]). Depending on the timing of ART initiation, some of these processes are not prevented, and in spite of low viral loads and normal CD4^+^ T cell counts, young individuals experience aging-related immune complications [3]. One study reported that HIV-infected persons on ART with a median age of 56 years, robust immune reconstitution and viral suppression had T cell characteristics similar to those of a group of HIV-uninfected subjects with a median age of 88 years (reviewed in [4]). T cell senescence is typically characterized by the accumulation of terminally differentiated T cells with shortened telomeres, loss of the costimulatory molecule CD28, and increased expression of CD57, a marker of poor proliferative capacity [5].

p16^INK4a^ protein (hereafter denoted as p16) is a mediator of cellular senescence and has been suggested to be a biomarker of ‘molecular’ age in several tissues including T cells [6], decreasing their replicative capacity [7]. Retinoblastoma protein (pRb) phosphorylation and concomitant entry into the cell division cycle can be prevented by the presence of the p16 protein, a cyclin-dependent kinase (CDK) inhibitor belonging to the INK4 family. It has been reported that p16 expression can be directly induced as a consequence of T cell activation [8]. Importantly, accumulation of p16 is responsible for the exit of a significant proportion of CD8^+^ T cells from the proliferative population, thus limiting their numerical expansion *in vitro* [9]. Results from disparate genetic systems suggest that expression of p16 contributes to the manifestations of human aging, possibly through the promotion of senescence *in vivo*. Ultimately, if validated and correlated with other markers of immune health and function, p16 levels could be used to identify HIV+ patients with premature immune aging, identify risk factors for premature loss of immune function, and to test therapeutic strategies to prevent or reverse HIV-induced immunosenescence [10]. Given that chronic HIV infection induces several features of immunosenescence [1] we investigated differences in p16 protein expression in memory T cell subsets in a cohort of HIV-infected patients on and off ART, as well as age-matched healthy controls. In summary, our data indicate that HIV accelerates immune aging in infected subjects as measured by cellular p16 expression, and that ART is able to restore this trend to normal levels in the CD4 compartment. The increased p16 expression in the T_EM_ subset observed in infected individuals may contribute to effector function impairment during HIV infection and could be a potential target for therapeutic blockade.

## Materials and Methods

### Study Subjects

Samples of cryopreserved peripheral blood mononuclear cells (PBMCs) were selected from participants in the San Francisco-based HIV-1-infected SCOPE cohort. Samples from HIV-1-seronegative controls were obtained from 19 donors of the Stanford blood bank. The study was approved by the local Institutional Review Board (University of California San Francisco Committee on Human Research) and individuals gave written informed consent. PBMCs were obtained from the following categories of HIV-1-infected individuals:

Untreated HIV subjects:

29 untreated virologic “Controllers” (C),
28 untreated “virologic Non-Controllers” (NC)
29 untreated “Immunologic Progressors” (IP)

Treated HIV subjects

27 ART-suppressed patients (HS)

All groups presented CD4^+^ T cell counts >250 cells/mm^3^ (C, NC and HS), except the IP group with CD4^+^ T cell counts <250 cells/mm^3^. All patients had been diagnosed with HIV-1 at least one year prior to inclusion in this study. See Table 1 for baseline subject characteristics.

**Table 1:** Clinical data.

	CD4^+^ count (cells/mm^3^) Median (IQR)	HIV-1 Viral load (copies/ml) Median (IQR)	Age (years) Median (IQR)
**HAART-suppressed (HS)**	674 (534–981)	50 (0–75)	45 (38–54)
**Controllers (C)**	813 (585–1260)	96 (11–489)	47.5 (39–54)
**Non-controllers (NC)**	576 (446–730)	35,650 (24,586–52675)	43 (34–49)
**Immunologic Progressors (IP)**	228 (175–296)	71,585 (29,925–191,067)	41.5 (36–49)
**Healthy Controls (HC)**	NA	NA	49 (45–53)

IQR = interquartile range, NA = non-applicable

### Antibodies and flow cytometry

Cryopreserved PBMC were thawed in RPMI 1640/10% FBS and washed in FACS buffer. Phenotypic staining was performed on a million cells by incubation with a viability marker (live-dead kit-Invitrogen) and with antibodies conjugated to CD3, CD4, CD8, CD45RA, CCR7, CD27, CD38 and CD95 (BD Biosciences, San Diego, CA) for 30 minutes on ice. Subsequently, cells were washed, fixed with paraformaldehyde 4% in PBS, washed and then permeabilized/stained for intracellular p16 (BD Biosciences, San Diego, CA, p16INK4 mAb (Mouse IgG1) PE-set, clone: G175-1239) with 0.2% saponine for 30 minutes at room temperature. Cells were then washed, fixed and resuspended in 150uL Macs buffer for acquisition in LSR-II flow cytometer (Becton Dickinson). After acquisition the data was analysed using the Flow Jo Software. The gate strategy was as followed: Live singlet CD4^+^ or CD8^+^ T cells were selected. In the CD3^+^CD4^+^ or CD3^+^CD8^+^ populations the MFI of p16+ were evaluated. The p16 gating strategy was based on fluorescence minus one (FMO) staining and the median intensity of fluorescence (MFI) was calculated in the p16^+^ gate. To discriminate memory subsets, CD45RA, CCR7 and CD27 markers were utilized. Central memory (T_CM_) was defined as CD45RA^-^CCR7^+^CD27^+^ and effector memory (T_EM_) as CD45RA-CCR7-CD27-. CD95 was used to discriminate naive and stem cell memory (T_SCM_). Naive cells were defined as CD45RA^+^CCR7^+^CD27^+^CD95^-^ and T_SCM_ as CD45RA^+^CCR7^+^CD27^+^CD95^+^.

### Statistical Analysis

Statistical analysis was performed using GraphPad Prism statistical software, version 6b (GraphPad Software, San Diego, CA). Non-parametric Kruskal-Wallis and Mann-Whitney U tests were used for group comparisons. Dunn’s post-hoc test (which incorporates the Bonferroni adjustment to correct for multiple comparisons) was used for between-group analyses. The Spearman rank test with linear regression was used for correlation analyses. P-values less than 0.05 were considered significant.

## Results

**Fig 1:**
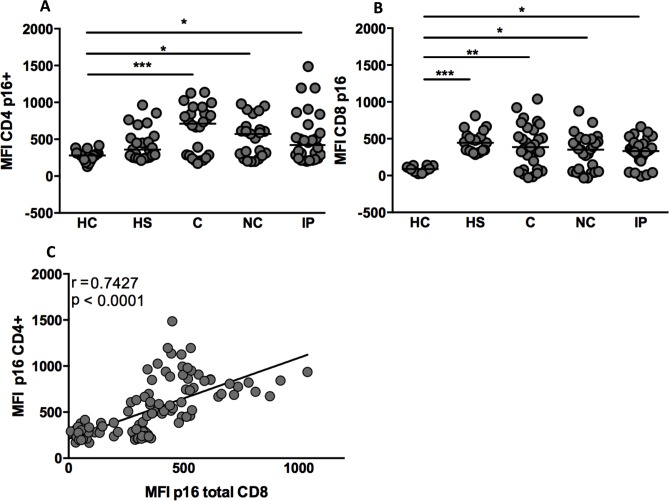
HIV drives higher per-cell p16 protein levels in CD4^+^ and CD8^+^ T lymphocytes among HIV-1-infected subjects. PBMC from healthy controls (HC), HAART suppressed (HS), Controllers (C), Immunological progressors (IP) and Non-controllers (NC) were stained for surface markers as well as the intracellular p16 marker. The median intensity of fluorescence (MFI) showing per-cell level expression of p16 in CD4^+^ and CD8 T^+^ cells is also shown (A and B, respectively). (C) Correlation between p16 MFI in CD4^+^ and CD8^+^ T cells in the whole cohort (Spearman rho, r = 0.74/p < 0.0001). *p<0.05, **p<0.01, ***p<0.001, ****p<0.0001.

Clinical characteristics of our cohort are detailed in Table 1. The enrolled subjects were divided in the following groups: A) Untreated HIV-1 infected subjects (1) HIV controllers (C) (n = 29), (2) early non-controllers (NC) (n = 28), (3) late non-controllers, called immunological progressors (IP)(n = 29) and B) Treated HIV-1 infected subjects (4) ART-suppressed (HS) (on treatment, n = 27). We also included a cohort of HIV-uninfected controls (HC) (n = 19). All subjects were adults with an average age of 49 years. ART-suppressed and controller individuals have preserved CD4^+^ T cell counts and low viral loads while early non-controllers and late non-controller groups have high viral loads, but are distinguished by their CD4^+^ T cell counts (median 611 vs. 228 cells/mm^3^, respectively). To define the immune aging profile of our cohort we analysed the per-cell level expression of p16 (median fluorescence intensity, MFI). All HIV-infected subjects had higher levels of p16 than HC in the CD4^+^ and CD8^+^ T cell compartments (CD4 subset—MFI: 280, 359, 712, 572 and 422 for HC, HS, C, NC and IP, respectively, with p<0.001 and <0.05 for HC *versus* C and HC *versus* NC/IP, respectively; CD8 subset–MFI: 88, 445, 387, 352 and 333 for HC, HS, C, NC and IP, respectively, with p<0.001, <0.01 and <0.05 for HC *versus* HS, C and NC/IP, respectively; Fig 1A and 1B, respectively). Nevertheless, CD4+ T cells from HS had the same p16 per-cell level as healthy controls. However, ART did not affect the p16 levels in CD8 T cells in the HS group. The p16 levels (MFI) for the whole cohort in CD4+ and CD8+ T cells were strongly correlated as shown in Fig 1C (r = 0.74, p < 0.0001).

**Fig 2:**
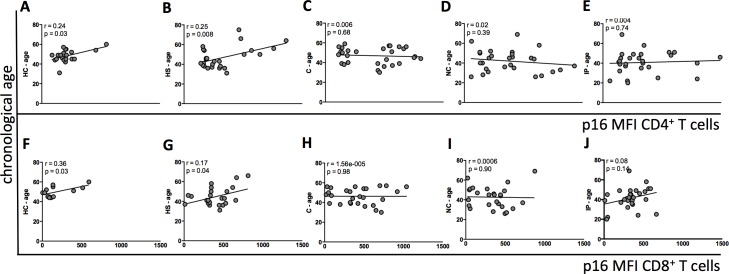
Chronological age and p16 expression in CD4^+^ and CD8^+^ T cells. Correlation between chronological age and per-cell level of p16 expression in each subject group is shown. (A-E) Correlation between chronological age in HC, HS, C, IP, NC and MFI p16 CD4^+^, respectively; (F-J). Correlation between chronological age in HC, HS, C, IP, NC and MFI p16 CD8^+^, respectively. HC: Healthy controls; HS: HAART suppressed; C: controllers; IP: Immunological progressors; NC: non-controllers. p and Spearman rho (r) values are shown for each plot.

Next, we evaluated p16 protein expression in CD4^+^ and CD8^+^ T cells in all groups and correlated it with chronological age. In the HC and HS groups age was positively correlated with CD4^+^ and CD8^+^ p16 levels (MFI) (Fig 2A, 2B, 2F and 2G, respectively). However, this correlation was absent in the untreated HIV-infected subjects (Fig 2C, 2D, 2E, 2H, 2I and 2J).

**Fig 3:**
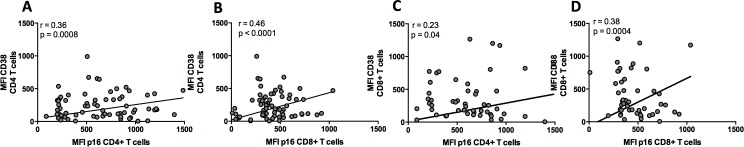
p16 expression is positively correlated with T cell activation. (A-B) Correlation between CD4^+^ T cell activation and p16 levels on CD4^+^ and CD8^+^, respectively; (C-D) Correlation between CD8^+^ T cell activation and p16 levels on CD4^+^ and CD8^+^, respectively. R and p values are shown for each plot.

We next analyzed the relationship between p16 MFI and cellular activation as measured by CD38 expression (MFI). We excluded the ART-suppressed group from these analyses to avoid the confounding effect of treatment on cellular activation and p16 levels. We observed a positive correlation between p16 levels in CD4+ and CD8+ T cells and CD38 expression in both subsets, indicating that immune aging is strongly correlated with immune activation (Fig 3A–3D).

**Fig 4:**
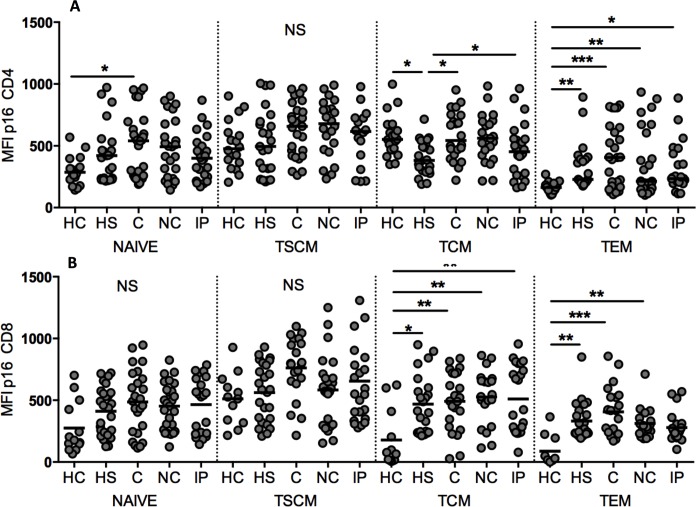
p16 expression in memory T cell subsets. (A and B) p16 MFI in CD4^+^ and CD8^+^ memory subsets, respectively, for all groups tested. HC: Healthy controls; HS: HAART suppressed; C: controllers; IP: Immunological progressors; NC: non-controllers. T_CM_: central memory T cells; T_SCM_: stem cell memory T cells, T_EM_: effector memory T cells. *p<0.05; **p<0.01; ***p<0.001; ****p<0.0001.

Finally, p16 per-cell expression in CD4^+^ and CD8^+^ memory T cell subsets was analyzed. The Fig 4A showed that p16 levels in CD4^+^ naïve and T_SCM_ were similar among all subjects (except the naive compartment in which the C group had higher p16 levels than HC). CD4^+^ T_CM_ from ART-suppressed subjects expressed lower levels of p16 than HC, C and IP subjects. Within the T_EM_ subset, p16 levels among HIV infected subjects were higher than the HC group. P16 levels in CD8^+^ naïve and T_SCM_ expressed similar p16 levels among all subjects (Fig 4B). In the T_CM_ subset all HIV infected groups expressed higher p16 levels than HC subjects. Similar trends were observed in the T_EM_ compartment suggesting that HIV infection leads to more senescent memory CD8^+^ T cells compared to uninfected subjects.

## Discussion

Expression of the cyclin-dependent kinase inhibitor p16 increases with age, and high levels of p16 result in cell cycle arrest [6]. In the present study we evaluated the levels of the p16 protein in total and memory CD4^+^ and CD8^+^ T cell subsets of HIV-infected subjects on and off ART as well as in age-matched healthy controls. Antiretroviral treated patients had p16 levels in CD4^+^ T cells comparable to those of uninfected individuals, particularly in the T_CM_ subset, suggesting that perhaps ART can restore the proliferative capacity of these cells to normal levels. Functional assays were not performed in this study due to limited cell numbers. Thus, questions remain regarding the threshold of p16 expression required to drive a cell into irreversible cell cycle arrest. The mechanism leading to disparate p16 expression among various T cell subsets as well as the direct effect of the virus on cellular p16 also remain to be determined.

Although the level of p16 per cell that can lead to cell cycle arrest is not known, its higher cellular expression in HIV-infected subjects is likely to impact the proliferative capacity of effector cells as well as other T cell subsets, globally impairing the ability to respond to the infection. Additionally, the non-effect of ART on restoring the p16 levels on CD8^+^ T cells might be an indicative that these cells already reached an irreversible activation/exhaustion status and the time-line to start ART might be important to restore the CD8+ T cells effector function and impact HIV infection outcome.

RT-PCR measurements have shown that p16 expression in total CD3^+^ cells is positively correlated with chronological aging in healthy individuals. This correlation is lost in untreated HIV-infected subjects but restored by ART, suggesting a role for viral replication in accelerating the aging process [6]. Our data corroborates this finding at the protein level, where p16 expression in both CD4^+^ and CD8^+^ T lymphocytes was positively correlated with age in healthy controls and ART-suppressed subjects but not maintained in the untreated HIV-infected group. Thus, ART can be effective in reestablishing the relationship between chronological and immunological aging in infected individuals.

We also found that the activation profile of total CD4^+^ and CD8^+^ T cells was positively correlated to p16 levels in CD4^+^ and CD8^+^ T lymphocytes, indicating that these parameters are likely linked in a pathway of cell activation and senescence. Future investigations of provirus load in p16-expressing cells could offer more direct insight into how viral infection can mediate senescence/activation.

Migliaccio et al. [9], reported that long term mitogen *in vitro* stimulation drives early naïve and T_CM_ proliferation that becomes quiescent after some rounds of division as the levels of p16 expression in these T cell compartments increase with time. The opposite dynamic was observed in T_EM_ cells. However, to our knowledge, the *ex vivo* p16 protein expression in memory T cell subsets has not been investigated previously. In the present work, *ex vivo* analysis of p16 expression revealed that CD4^+^ T_EM_ cells from healthy subjects have the lowest levels of p16 likely reflecting the ability of these cells to rapidly proliferate upon antigen stimulation. The highest amounts of p16 were found in CD4^+^ and CD8^+^ T_SCM_ subset as well as CD4^+^ T_CM_ cells, which are long-lived cells and provide lasting immunologic memory. Roederer et al., [11] have shown that T_SCM_ cells have the ability to proliferate after long term *in vitro* culture with IL-2 and IL-15, giving rise to the other memory subsets. It is possible that the requirement for a long-term stimulus to induce robust proliferation may be due to the high p16 expression in this T cell subset. CD4^+^ T_EM_ cells from all HIV-infected subjects had higher levels of p16 expression than healthy controls. However, subjects on ART had a trend towards lower levels of p16 in the CD4^+^ T_EM_ population and a significantly lower level in the T_CM_ cells than untreated individuals, indicating that virologic suppression with ART has an impact on p16 levels and also in the control of T cell cycling. As CD4^+^ T cells are critical in the orchestration of the immune response to pathogens by providing help to CD8^+^ cytotoxic T cells as well as to antibody secreting B cells, robust restoration of this population is fundamental and can have an impact on the HIV-associated morbidity. This may be one mechanism by which early HIV diagnosis and treatment can lead to more effective proliferating cells and immune restoration. Furthermore, targeting the p16 pathway may represent a future opportunity for immunotherapeutic intervention in chronic HIV infection.

## Abbreviations

T_SCM_: Stem memory T cell
T_EM_: Effector memory T cell
T_CM_: Central memory T cell
ART: Antiretroviral therapy
