# Correction: p16^INK4a^ Expression and Immunologic Aging in Chronic HIV Infection

**DOI:** 10.1371/journal.pone.0176509

**Published:** 2017-04-20

**Authors:** Susan Pereira Ribeiro, Jeffrey M. Milush, Edecio Cunha-Neto, Esper G. Kallas, Jorge Kalil, Luiz Felipe D. Passero, Peter W. Hunt, Steven G. Deeks, Douglas F. Nixon, Devi SenGupta

There is an error in the XML that is causing the first, second, fourth, sixth, seventh, eighth, and ninth authors’ names to be indexed incorrectly. The names should be indexed as: Ribeiro SP, Milush JM, Kallas EG, Passero LF, Hunt PW, Deeks SG, and Nixon DF.

The citation is incorrect in the published article. The correct citation is: Ribeiro SP, Milush JM, Cunha-Neto E, Kallas EG, Kalil J, Passero LF et al. (2016) p16^INK4a^ Expression and Immunologic Aging in Chronic HIV Infection. PLoS ONE 11(11): e0166759. doi:10.1371/journal.pone.0166759.
